# Assessment of Long‐Lasting Insecticide‐Treated Bed Net Possession, Utilization, and Prevalence of Malaria Among Patients Visiting Bahir Dar Health Center

**DOI:** 10.1155/japr/9689904

**Published:** 2026-06-17

**Authors:** Elsabet Adugna Gudeta, Daniel Mulu Mengistu, Sissay Menkir Mekonnen

**Affiliations:** ^1^ Department of Biology, College of Natural and Computational Sciences, Wachemo University, Hosaena, Ethiopia, wcu.edu.et; ^2^ Department of Biotechnology, College of Natural and Computational Sciences, Wachemo University, Hosaena, Ethiopia, wcu.edu.et; ^3^ Department of Biology, College of Natural and Computational Sciences, Bahir Dar University, Bahir Dar, Ethiopia, bdu.edu.et

**Keywords:** LLINs, malaria, *Plasmodium falciparum*, *Plasmodium vivax*, prevalence, vector control

## Abstract

Malaria remains a major public health problem in Ethiopia despite recent progress in control and elimination efforts, including the scale‐up of long‐lasting insecticidal nets (LLINs). A facility‐based cross‐sectional study was conducted from February to March 30, 2021, among suspected malaria patients attending Bahir Dar Health Center, Amhara Regional State, Northwest Ethiopia. A total of 403 participants were selected by simple random sampling. Data on sociodemographic characteristics and LLIN possession and utilization were collected using a structured questionnaire, and malaria infection was determined by microscopic examination of blood smears. Among a total of 403 participants, 283 (70.2%) possessed at least one LLIN and 268 (66.5%) reported regular use during sleeping hours. The overall malaria prevalence was 3.7% (15/403). *Plasmodium falciparum* was the predominant species (60.0%), followed by *Plasmodium vivax* (26.7%) and mixed infections (13.3%). Malaria prevalence was significantly higher among rural than urban residents (14.3% vs. 2.9%; *p* = 0.002) and among participants without LLINs compared with LLIN owners (8.0% vs. 1.8%; *p* = 0.002). No significant associations were observed between malaria prevalence and sex, age, educational status, or LLIN washing practices. Malaria transmission persists at measurable levels in Bahir Dar despite moderate LLIN coverage and utilization. Strengthening universal LLIN coverage, promoting proper and consistent use, and implementing targeted interventions in rural communities are essential to further reduce malaria transmission.

## 1. Introduction

Malaria remains one of the most significant public health problems worldwide, particularly in sub‐Saharan Africa, where it continues to cause substantial morbidity and mortality [1, 2]. The disease is caused by protozoan parasites of the genus *Plasmodium* and is transmitted through the bites of infected female *Anopheles* mosquitoes. Five *Plasmodium* species are known to infect humans, including *Plasmodium falciparum*, *Plasmodium vivax*, *Plasmodium ovale*, *Plasmodium malariae*, and *Plasmodium knowlesi*. Among these, *P. falciparum* is considered the most virulent malaria parasite species and is responsible for the majority of severe malaria cases and malaria‐related deaths worldwide. In contrast, *P. vivax*, *P. ovale*, and *P. malariae* generally cause comparatively milder forms of malaria, while *P. knowlesi* contributes less frequently to human malaria infections [3, 4]. Clinical manifestations of malaria commonly include high fever, chills, headache, sweating, fatigue, and flu‐like symptoms, which may progress to severe complications if not properly managed [5, 6].

According to the World Health Organization (WHO), an estimated 229 million malaria cases and 409,000 malaria‐related deaths occurred globally in 2019. Children under 5 years of age remain the most vulnerable group, accounting for approximately 67% of global malaria deaths. The WHO African Region bears the greatest burden of malaria, contributing nearly 94% of all malaria cases and deaths worldwide [7]. Ethiopia is among the malaria‐endemic countries in Africa, where malaria continues to pose a major public health challenge [8, 9]. Approximately 75% of the country’s landmass is considered a malaria‐risk area, and nearly 68% of the population lives in areas vulnerable to malaria transmission [8]. In Ethiopia, *P. falciparum* and *P. vivax* are the predominant malaria parasite species, accounting for the majority of malaria infections. National malaria reports have documented millions of confirmed malaria cases and substantial malaria‐associated mortality over recent years [10]. Although considerable progress has been achieved through national malaria control programs, malaria outbreaks continue to occur in different parts of the country [11].

Malaria transmission in Ethiopia is highly influenced by climatic and ecological factors, particularly altitude, rainfall, humidity, and temperature [12]. The major malaria transmission season commonly occurs between September and December following the main rainy season, while a secondary transmission period occurs between April and May after the short rainy season [3]. Bahir Dar and its surrounding areas possess environmental and ecological conditions favorable for malaria transmission, including proximity to Lake Tana, seasonal rainfall patterns, and suitable mosquito breeding habitats. A previous geographical information system (GIS)–based malaria risk mapping study conducted in Bahir Dar reported the presence of high‐, moderate‐, and low‐risk malaria transmission zones within the city and surrounding areas [13]. Long‐lasting insecticidal nets (LLINs) are among the most effective malaria prevention interventions and remain a cornerstone of malaria control strategies in Ethiopia. The Ethiopian government has implemented large‐scale LLIN distribution programs aimed at reducing malaria transmission and malaria‐related mortality. However, the effectiveness of LLINs depends not only on ownership but also on consistent and proper utilization at the household level. Previous studies conducted in Ethiopia have primarily focused on malaria prevalence, retrospective trend analyses, or community‐level LLIN ownership and distribution separately [12].

In addition, many earlier studies relied mainly on questionnaire‐based assessments without simultaneously incorporating laboratory‐confirmed malaria diagnosis to evaluate the direct relationship between LLIN utilization practices and malaria infection status. Furthermore, limited recent health facility–based studies have comprehensively assessed LLIN possession and utilization practices and parasitologically confirmed malaria prevalence within the same study population in the Bahir Dar area. Most existing studies also provide insufficient evidence regarding how sociodemographic factors, household LLIN utilization behaviors, and environmental conditions collectively influence malaria prevalence in this ecological setting [13]. This represents an important knowledge gap for malaria prevention and control planning in the region. Therefore, this study was conducted to assess LLIN possession, utilization practices, and malaria prevalence among suspected malaria patients attending Bahir Dar Health Center, Northwest Ethiopia. The findings of this study are expected to provide updated evidence that may support ongoing malaria control strategies and targeted intervention programs in the study area and similar malaria‐endemic settings.

## 2. Materials and Methods

### 2.1. Description of the Study Area

The study was conducted at Bahir Dar Health Center, located in Fasilo Subcity, Bahir Dar City, Amhara National Regional State, Northwest Ethiopia. Bahir Dar is situated approximately 565 km northwest of Addis Ababa, the capital city of Ethiopia. Geographically, the city lies between 11^°^33^′^15^″^ and 11^°^36^′^53^″^ North latitude and 37^°^21^′^11^″^ and 37^°^25^′^49^″^ East longitude. Bahir Dar has served as the capital city of the Amhara National Regional State since 1991 and currently covers an area of approximately 256.4 km^2^. According to the 2007 national census report, the city had a total population of 221,991, comprising 108,456 males and 113,535 females [14]. The city is located at an altitude ranging from 1783 to 1889 m above sea level and lies near the southern shore of Lake Tana, the largest freshwater lake in Ethiopia and the source of the Blue Nile River. Bahir Dar experiences a tropical climate characterized by annual rainfall averaging 1409 mm and mean temperatures ranging from 21°C to 30°C. The major rainy season occurs from June to August, while a short rainy season occurs between February and March [6, 15]. Previous malaria risk mapping studies conducted in Bahir Dar have identified areas with varying levels of malaria transmission risk, ranging from low to high endemicity [13].

### 2.2. Study Design

A health facility–based cross‐sectional study was conducted among malaria‐suspected patients attending Bahir Dar Health Center, Northwest Ethiopia, from February to March 2021. The study employed structured questionnaires and laboratory‐based parasitological examination to assess LLIN possession, utilization practices, and malaria prevalence.

### 2.3. Inclusion and Exclusion Criteria

Participants who visited Bahir Dar Health Center during the study period, provided blood samples for malaria diagnosis, and consented to participate in the study were included. Individuals who had taken antimalarial drugs within 2 weeks prior to data collection, declined blood examination, or were unwilling to participate in the questionnaire survey were excluded from the study.

### 2.4. Sample Size Determination

The sample size was determined using the single population proportion formula for estimating a proportion in a cross‐sectional study.

n=Z2P1−Pd2,

where *n* = required sample size, *Z* = standard normal distribution value at a 95*%*confidence interval (1.96), *P* = estimated prevalence, and *d* = margin of error (0.05) [16]. Since there was no previously published study specifically reporting the prevalence of malaria and LLIN utilization among suspected malaria patients in the study area, a prevalence estimate of 50% was used to obtain the maximum sample size [17]. Using a 95% confidence level and a 5% margin of error, the calculated minimum sample size was 384 participants. After adding 5% to compensate for possible nonresponse, the final sample size became 403 participants.

### 2.5. Questionnaire Survey

A structured questionnaire was developed after reviewing standardized malaria indicator survey tools and previously published studies related to LLIN utilization and malaria prevention [18]. The questionnaire included sections addressing sociodemographic characteristics, LLIN ownership, frequency of utilization, sleeping practices, net maintenance behavior, and environmental risk factors associated with malaria transmission. The questionnaire was initially prepared in English, translated into Amharic, and then back‐translated into English to ensure consistency of meaning. Prior to the actual data collection, the questionnaire was pretested on 5% of participants outside the study area to evaluate clarity, reliability, and appropriateness. Necessary modifications were made based on the pretest findings.

### 2.6. Laboratory Blood Film Collection and Testing

Blood samples were collected by a trained laboratory technician through finger‐prick procedures from participants suspected of malaria during clinical examination. Both thick and thin blood smears were prepared on clean glass slides for microscopic diagnosis. The prepared smears were properly labeled and allowed to air‐dry. Thin blood films were fixed with methanol for 15 s, whereas thick smears were left unfixed. The blood smears were stained using 10% Giemsa solution for 30 min following standard WHO malaria microscopy procedures [6, 19]. After staining, the slides were gently rinsed with clean tap water, air‐dried, and examined under a light microscope using a 100× oil immersion objective lens [16]. Thick blood films were used for detection of malaria parasites, while thin blood films were used for identification of *Plasmodium* species. All laboratory procedures and slide examinations were performed according to WHO‐recommended malaria diagnostic guidelines.

### 2.7. Data Analysis

After checking for completeness and consistency, the data were coded and entered into SPSS Version 25 for analysis. Descriptive statistics were used to summarize the data. Associations between categorical variables and malaria prevalence were assessed using chi‐square tests. Fisher’s exact test was applied when expected cell counts were less than five. Statistical significance was considered at *p* < 0.05.

### 2.8. Data Quality Control

To ensure data quality and consistency, the questionnaire was initially prepared in English and subsequently translated into Amharic, the local language of the study area. The translated version was then back‐translated into English to verify consistency and minimize distortion of meaning. Prior to the actual data collection, the questionnaire was pretested on 5% of the study participants in health facilities outside the study area to evaluate the clarity, reliability, and applicability of the questions. Necessary modifications were made based on the pretest findings. Standard operating procedures were followed during blood sample collection, smear preparation, staining, and microscopic examination. All laboratory results were carefully recorded using standardized data recording formats. Data completeness and consistency were checked daily during the data collection process before data entry and analysis.

### 2.9. Ethical Consideration

Ethical clearance was obtained from the Ethical Clearance Committee of the College of Science, Bahir Dar University, prior to commencement of the study. Written informed consent was obtained from all adult participants after explaining the purpose and procedures of the study. Participation was entirely voluntary, and confidentiality of participant information was maintained throughout the study. For participants under 18 years of age, written informed consent was obtained from parents or legal guardians before enrollment in the study.

## 3. Results

### 3.1. Sociodemographic Characteristics of Study Participants

A total of 403 participants were included in the study. Of these, 228 (56.6%) were males and 175 (43.4%) were females. Regarding age distribution, 124 (30.8%) participants were between 10 and 25 years of age, followed by 107 (26.6%) participants aged 26–40 years. Children under 5 years accounted for 103 (25.6%) of the participants, while 48 (11.9%) were above 40 years of age. Most participants, 375 (93.1%), were urban residents, whereas 28 (6.9%) lived in rural areas. Concerning family size, 222 (55.1%) participants belonged to households with 2–5 family members, while 67 (16.6%) had family sizes of 6–10 members. With respect to educational status, 105 (26.1%) participants were illiterate, 98 (24.3%) had attended high school, and 95 (23.6%) had elementary‐level education. Participants with diploma and degree‐level education accounted for 55 (13.6%) and 50 (12.4%), respectively. Regarding occupational status, 153 (38.0%) participants were employed in the private sector, 79 (19.6%) were unemployed, and 72 (17.9%) were government employees. In terms of duration of residence in the study area, 128 (31.8%) participants had lived in the area since birth, while 106 (26.3%) had resided in the area for < 5 years (Table 1).

**Table 1:** Sociodemographic characteristics of respondents.

Characteristics		Frequency	Percentage (%)
Gender	Female	175	43.4
	Male	228	56.6

Age	< 5 years	103	25.6
	6–9 years	21	5.2
	10–25 years	124	30.8
	26–40 years	107	26.6
	> 40 years	48	11.9
	Total	403	100

Home residence	Rural	28	6.9
	Urban	375	93.1
	Total	403	100

Family size	Single	114	28.3
	2–5 members	222	55.1
	6–10 members	67	16.6
	Total	403	100

Educational status	Illiterate	105	26.1
	Elementary	95	23.6
	High school	98	24.3
	Diploma	55	13.6
	Degree and above	50	12.4
	Total	403	100

Occupational status	Unemployed	79	19.6
	Farmer	65	16.1
	Private sector	153	38.0
	Governmental	72	17.9
	Student	34	8.4
	Total	403	100

Duration of residence in the area	< 5 years	106	26.3
	5–10 years	82	20.3
	Above 10 years	87	21.6
	Since birth	128	31.8
	Total	403	100

### 3.2. LLIN Possession and Utilization

Among the 403 study participants, 283 (70.2%) reported ownership of at least one LLIN, while 120 (29.8%) did not possess any LLINs. Regarding the source of LLINs, 155 (38.5%) participants obtained the nets through governmental distribution programs, 113 (28.0%) purchased them privately, and 15 (3.7%) acquired LLINs from both sources. Concerning household LLIN availability, 168 (41.7%) participants reported having one LLIN, 81 (20.1%) had two LLINs, and 34 (8.4%) possessed three or more LLINs. Among LLIN owners, 268 (66.5%) reported using LLINs during sleeping time, whereas 15 (3.7%) reported not using them. Regarding frequency of utilization, 224 (55.6%) participants reported always using LLINs, while 44 (10.9%) reported occasional use. Assessment of household sharing indicated that 115 (28.5%) households shared one LLIN between two family members, whereas 109 (27.0%) households shared one LLIN among three or more members. Regarding maintenance practices, 238 (59.0%) participants reported washing LLINs when dirty, while 45 (11.2%) did not wash their nets. In addition, 203 (50.4%) participants reported that their LLINs were intact without holes, whereas 80 (19.8%) reported torn LLINs (Table 2).

**Table 2:** Ownership, possession, and utilization practices of long‐lasting insecticidal nets (LLINs) among study participants.

Variables	Responses	Frequency	Percentage (%)
Do you have an insecticide‐treated bed net?	Yes	283	70.2
	No	120	29.8
	Total	403	100.0

Where did you obtain the insecticide‐treated bed net?	Governmental	155	38.5
	Purchased	113	28.0
	Both	15	3.7
	Total	283	70.2

How many insecticide‐treated bed nets do you have?	One bed net	168	41.7
	Two bed nets	81	20.1
	Three and above	34	8.4
	Total	283	70.2

Do you use an insecticide‐treated bed net during sleeping time?	Yes	268	66.5
	No	15	3.7
	Total	283	70.2

How many members of the family use a single bed net?	Single member	44	10.9
	Two members	115	28.5
	Three and above	109	27.0
	Total	268	66.4

How often do you use an insecticide‐treated bed net?	Sometimes	44	10.9
	Always	224	55.6
	Total	268	66.5

Do you wash your bed net when it becomes dirty?	Yes	238	59.0
	No	45	11.2
	Total	283	70.2

Does your bed net have tears?	Yes	80	19.8
	No	203	50.4
	Total	283	70.2

### 3.3. Malaria Prevalence

The overall malaria prevalence among study participants was 3.7% (15/403). Malaria prevalence was significantly higher among rural residents than urban residents (14.3% vs. 2.9%; *p* = 0.002). Participants who did not possess LLINs had significantly higher malaria prevalence compared to LLIN owners (8.0% vs. 1.8%; *p* = 0.002). No statistically significant associations were observed between malaria prevalence and sex, age group, or educational status (Table 3).

**Table 3:** Prevalence of malaria infection according to sociodemographic characteristics among study participants.

Variables		Test for malaria cases			
		Positive *N* (%)	Negative *N* (%)	*χ* ^2^(df)	*p* value
Sex	Male	9 (4.0)	219 (52.1)	0.074^a^ (1)	0.785
	Female	6 (3.4)	169 (96.5)		

Residential place	Urban	11 (2.9)	364 (97.1)	9.370^a^ (1)	0.002 ^∗^
	Rural	4 (14.3)	24 (85.7)		

Educational status	Illiterate	5 (4.8)	100 (95.2)	0.059 (4)	0.847
	Elementary	4 (4.2)	91 (95.8)		
	High school	4 (4.1)	94 (95.9)		
	Diploma	1 (1.8)	54 (98.2)		
	Degree and above	1 (2.0)	49 (98.0)		

Age group	< 5 years	3 (2.9)	100 (97.1)	0.028 (4)	0.989
	6–9 years	1 (4.8)	20 (95.2)		
	10–25 years	5 (4.0)	119 (96.0)		
	26–40 years	4 (3.7)	103 (96.3)		
	> 40 years	2 (4.2)	46 (95.8)		

LLIN possession	Yes	5 (1.79)	278 (98.23)	9.373	0.002 ^∗^
	No	10 (8.0)	110 (91.67)		

Abbreviation: df, degree of freedom.

^∗^Statistically significant at *p* < 0.05.

### 3.4. Distribution of *Plasmodium* Species

Among the 15 malaria‐positive participants, *P. falciparum* was the predominant species, accounting for nine cases (60.0%), followed by *P. vivax* with four cases (26.7%). Mixed infection with both *P. falciparum* and *P. vivax* was identified in two participants (13.3%) (Table 4). Analysis by sex showed that among male participants, six cases (66.7%) were infected with *P. falciparum* and one case (25.0%) with *P. vivax*, and two cases (100.0%) had mixed infections. Among female participants, three cases (33.3%) were infected with *P. falciparum* and three cases (75.0%) with *P. vivax*, while no mixed infections were observed among females. No statistically significant association was observed between sex and *Plasmodium* species distribution (*χ* ^2^ = 4.309, df = 1, *p* = 0.230). Age‐specific analysis indicated that *P. falciparum* infections occurred across most age groups, whereas *P. vivax* infections were mainly observed among children and young adults. Mixed infections were identified in the 10–25‐ and 26–40‐year age groups. However, there was no statistically significant association between age group and *Plasmodium* species distribution (*χ* ^2^ = 10.328, df = 4, *p* = 0.587). Place of residence showed a statistically significant association with *Plasmodium* species distribution (*χ* ^2^ = 12.206, df = 1, *p* = 0.002). Among urban residents, six cases (66.7%) were caused by *P. falciparum* and three cases (75.0%) by *P. vivax*, and both mixed infections were recorded in urban participants. In rural residents, three cases (33.3%) were due to *P. falciparum* and one case (25.0%) due to *P. vivax*.

**Table 4:** Distribution of *Plasmodium* species among malaria‐positive participants according to sociodemographic characteristics.

		Predominance of *Plasmodium* species			*ꭓ* ^2^(df)	*p* value
		*P. falciparum*	*P. vivax*	Mixed species		
Sex	Male	6 (66.7)	1 (25.0)	2 (100.0)	4.309 (1)	0.230
	Female	3 (33.3)	3 (75.0)	0 (0.0)		

Age group	< 5 years	1 (11.1)	2 (50.0)	0 (0.0)	10.328 (4)	0.587
	6–9 years	0 (0.0)	1 (25.0)	0 (0.0)		
	10–25 years	3 (33.3)	1 (25.0)	1 (25.0)		
	26–40 years	3 (33.3)	0 (0.0)	1 (25.0)		
	> 40 years	2 (22.2)	0 (0.0)	0 (0.0)		

Residential place	Urban	6 (66.7)	3 (75.0)	2 (100.0)	12.206^a^ (1)	0.002 ^∗^
	Rural	3 (33.3)	1 (25.0)	0 (0.0)		

Abbreviation: df, degree of freedom.

^∗^Statistically significant at *p* < 0.05.

### 3.5. Sociodemographic Factors, LLIN Possession, and Utilization

Malaria prevalence showed a significant association with residential area and LLIN possession (Table 5). Rural residents had a higher malaria prevalence (14.3%) compared to urban residents (2.9%) (*χ* ^2^ = 9.370, df = 1, *p* = 0.002). No statistically significant association was observed between malaria prevalence and the presence of stagnant water near households (*χ* ^2^ = 0.008, df = 1, *p* = 0.931). Participants living near stagnant water had a malaria prevalence of 3.6%, whereas those without stagnant water nearby had a prevalence of 3.8%. The physical condition and utilization practices of LLINs were also not significantly associated with malaria prevalence. Malaria prevalence was 3.7% among participants whose nets had holes compared to 1.0% among those with intact nets (*χ* ^2^ = 2.278, df = 1, *p* = 0.133). Likewise, malaria prevalence among LLIN users was 1.5%, while nonusers had a prevalence of 7.1%, although the difference was not statistically significant (*χ* ^2^ = 2.667, df = 1, *p* = 0.100). Washing practice of LLINs also showed no significant association with malaria prevalence (*χ* ^2^ = 0.095, df = 1, *p* = 0.758).

**Table 5:** Association of sociodemographic factors, LLIN possession, and utilization practices with malaria prevalence among study participants.

Variables		Malaria prevalence		*χ* ^2^(df)	*p*value
		Positive (%)	Negative (%)		
Home residence	Urban	11 (2.9)	364 (97.1)	9.370^a^ (1)	0.002 ^∗^
	Rural	4 (14.3)	24 (85.7)		

Is there stagnant water in your residential area?	Yes	3 (3.6)	81 (96.4)	0.008^a^ (1)	0.931
	No	12 (3.8)	307 (96.2)		

Do you have LLINs?	Yes	5 (1.79)	273 (98.2)	9.373^a^ (1)	0.002 ^∗^
	No	10 (8)	115 (92)		

Does your bed net have any holes?	Yes	3 (3.65)	79 (96.35)	2.278^a^ (1)	0.133
	No	2 (1)	194 (99)		

Do you use LLINs during sleeping time?	Yes	4 (1.5)	260 (98.5)	2.667^a^ (1)	0.1
	No	1 (7.14)	13 (92.84)		

Do you wash your bed net?	Yes	4 (1.69)	232 (98.31)	0.095^a^ (1)	0.758
	No	11 (6.59)	156 (93.41)		

Abbreviation: df, degree of freedom.

^∗^Statistically significant at *p* < 0.05.

## 4. Discussions

This study assessed LLIN possession, utilization, and malaria prevalence among suspected malaria patients attending Bahir Dar Health Center. The overall malaria prevalence was 3.7%, which is lower than findings reported from other regions of Ethiopia, including studies conducted in Chagni and Goljota Health Centers [20]. The relatively lower prevalence observed in the present study may be associated with seasonal variation, ongoing malaria control interventions, and improved access to preventive measures such as LLINs [15, 19, 20]. LLIN ownership in this study was moderate, with approximately 70% of participants possessing at least one LLIN. Ownership of LLINs was significantly associated with reduced malaria prevalence, supporting evidence from previous studies demonstrating the protective effect of LLINs against malaria transmission [7, 21]. Participants without LLINs showed substantially higher malaria prevalence than LLIN owners, emphasizing the importance of expanding universal coverage.

Although regular LLIN utilization showed a lower malaria prevalence among users compared to nonusers, the association was not statistically significant. This may be explained by inconsistent utilization practices, improper net usage, household overcrowding, or deterioration in net quality over time. Similar observations have been reported in previous Ethiopian studies [16]. Residence was significantly associated with malaria prevalence, with rural residents exhibiting considerably higher infection rates than urban residents. Rural communities are often more exposed to mosquito breeding sites and may have reduced access to healthcare services and malaria prevention interventions [21–25]. The predominance of *P. falciparum* observed in this study is consistent with the epidemiological pattern reported in Ethiopia, where *P. falciparum* remains the leading cause of malaria morbidity and mortality [4, 19, 26–28]. The persistence of *P. falciparum* infections indicates the continued need for strengthened vector control and surveillance measures. Although sex, age, and educational status were not significantly associated with malaria prevalence, these factors remain epidemiologically relevant and may influence malaria exposure and prevention behaviors [29, 30]. The absence of significant associations for age, sex, and some LLIN utilization indicators may be related to the relatively low malaria prevalence observed during the study period.

## 5. Limitations

Although this study provides important baseline information regarding LLIN possession, utilization, and malaria prevalence among patients attending Bahir Dar Health Center, some limitations should be acknowledged. The assessment of LLIN utilization was based on participants’ self‐reported responses, which may be influenced by recall variation or reporting bias. In addition, the study was conducted at a single health facility using a cross‐sectional design; therefore, the findings primarily reflect the conditions during the study period and may not fully capture seasonal variations in malaria transmission. Moreover, entomological parameters such as mosquito vector density, insecticide resistance status, and LLIN bioefficacy were not included in the present investigation. Nevertheless, the study combined laboratory‐confirmed malaria diagnosis with household‐level LLIN utilization assessment, providing valuable evidence on malaria prevention practices and associated malaria burden in the study area. Future studies incorporating longitudinal follow‐up, community‐based sampling approaches, and entomological assessments would further strengthen understanding of malaria transmission dynamics and vector control effectiveness in the region.

## 6. Conclusions

This study demonstrated that malaria prevalence among patients attending Bahir Dar Health Center was relatively low; however, transmission persists, particularly in rural communities. Moderate LLIN ownership and utilization were observed among the study participants. Ownership of LLINs was significantly associated with reduced malaria prevalence, confirming the importance of LLINs as a major malaria prevention strategy. The predominance of *P. falciparum* indicates the continued public health importance of severe malaria in the study area. Rural residence was identified as a significant risk factor for malaria infection, suggesting the need for geographically targeted interventions. Overall, strengthening universal LLIN coverage, improving consistent utilization practices, and enhancing rural malaria control programs are essential for sustaining malaria prevention efforts and advancing toward malaria elimination goals in Ethiopia.

## Author Contributions

**Elsabet Adugna Gudeta:** conceptualization, data collection, data analysis, manuscript drafting. **Daniel Mulu Mengistu:** statistical analysis, manuscript revision, critical editing. **Sissay Menkir Mekonnen:** supervision, validation, manuscript review, and editing.

## Funding

No funding was received for this manuscript.

## Disclosure

A version of this manuscript was previously published as a thesis in the institutional repository [31]. All authors read and approved the final manuscript.

## Ethics Statement

Ethical clearance was obtained from the Ethical Clearance Committee of the College of Science, Bahir Dar University. Written informed consent was obtained from all adult participants prior to enrollment. For participants under 18 years of age, written consent was obtained from parents or legal guardians. The study was conducted in accordance with the Declaration of Helsinki.

## Conflicts of Interest

The authors declare no conflicts of interest.

## Data Availability

The datasets generated and analyzed during the current study are available from the corresponding author upon reasonable request.
